# Association of maternal infection of SARS-CoV-2 and neonatal susceptibility: A retrospective cohort study

**DOI:** 10.1016/j.jvacx.2024.100536

**Published:** 2024-07-26

**Authors:** Xiao-Dan Zhu, Yan-Jie Peng, Ying Chen, Mei Xue, Ai-Juan Zhang, Yu Peng, Rong Mei, Mei-Rong Tian, Lin Zhang

**Affiliations:** a Obstetrics Department, Shandong Provincial Maternal and Child Health Care Hospital Affiliated to Qingdao University, Jinan 250001, China; b Clinical Medical Research Center for Women and Children Diseases, Key Laboratory of Birth Regulation and Control Technology of National Health Commission of China Shandong Provincial Maternal and Child Health Care Hospital Affiliated to Qingdao University, Jinan 250001, China; c Key Laboratory of Birth Defect Prevention and Genetic Medicine of Shandong Health Commission, Jinan 250001, China; d Pediatric Surgery Department, Shandong Provincial Maternal and Child Health Care Hospital Affiliated to Qingdao University, Jinan 250001, China

**Keywords:** COVID-19 pandemic, Infection, Pregnancy, Neonatal susceptibility

## Abstract

**Objective:**

This study aims to assess the risk of neonatal susceptibility to COVID-19 among pregnant women.

**Methods:**

We conducted a retrospective cohort study involving 1089 pregnant women ≥28 weeks of gestational age, who were categorized into infected and uninfected groups. Data for all participants were collected through a comprehensive review of electronic medical records and follow-up phone calls. The primary outcome was neonatal infection with SARS-CoV-2, while secondary outcomes included delivery patterns and gestational age at delivery.

**Results:**

Maternal vaccination (OR 95%CI:0.63[0.46, 0.85]) and maternal infection with SARS-CoV-2 (OR 95%CI: 0.45[0.34, 0.60]) were found to be associated with a decreased risk of neonatal infection. The infected group exhibited a lower neonatal SARS-CoV-2 infection rate (25.93%) compared to the uninfected group (45.15%). Logistic regression analysis identified several risk factors associated with an increased risk of neonatal infection, including pregnancy BMI (OR 95%CI: 1.04[1.01, 1.08]), age at first pregnancy (OR 95%CI: 1.05[1.01, 1.10]), age at menarche (OR 95%CI: 1.13[1.02, 1.26]), and parturition (Yes vs. No) (OR 95%CI:1.4 [1.04,1.88]).

**Conclusion:**

Maternal vaccination and perinatal infection with SARS-CoV-2 play a protective role in preventing neonatal SARS-CoV-2 infection.

## 1 Introduction

Coronavirus disease 2019 (COVID-19) is a highly contagious respiratory disease caused by SARS-CoV-2, its most common clinical symptoms include fever, cough, fatigue, headache, dyspnea, and diarrhea [1]. The World Health Organization (WHO）declared the COVID-19 outbreak as the sixth public health emergency of international concern on January 30,2020 then proclaimed it a global pandemic on March 11, 2020[2], [3]. More than 15 million people have died in the pandemic [4]. When loosened the COVID-19 restrictions on December 7,2022 in China, a large influx of pregnant women infected with COVID-19 posed a huge challenge for obstetricians to ensure the safety of mothers and infants.

Due to changes in physical, physiological, and immunosuppressive status, pregnant women are particularly prone to respiratory infections and severe pneumonia [5]. The mortality rate of pregnant women infected with SARS-CoV-2 is as high as 0.9 %[6]. Some studies suggest that infection with SARS-CoV-2 during pregnancy is associated with many adverse pregnancy outcomes, including pre-eclampsia, premature birth, and stillbirth, especially in pregnant women with severe COVID-19 disease [7], [8], [9]. However, others indicate that maternal infection with SARS-CoV-2 during pregnancy usually do not have severe consequences on mother and child [10], [11], [12]. As mentioned above, most of the studies mainly focus on the maternal and fetal outcomes in COVID-19-positive pregnant women, there is scarcely research on the susceptibility of newborns with SARS-CoV-2, and therefore this comparative retrospective cohort study was conducted with the aim to identify the risk of maternal SARS-CoV-2 infection on neonatal susceptibility.

## 2 Methods

### 2.1 Data source and study design

This retrospective cohort study involved 1089 pregnant women who were hospitalized in Shandong Provincial Maternal and Child Health Care Hospital between November 7, 2022 and January 7,2023, one month before and after the easing of COVID-19 control measures in China. The cohort had been registered and named Maternal SARS-CoV-2 Infection and Offspring Susceptibility (MSIOS, clinical registration number: MR-37-23-019409). All patient data including demographic characteristics, medication, and laboratory examination results were obtained from reviewing electronic medical records. Additional information, such as COVID-19 symptoms in pregnant women, paternal baseline characteristics, and neonatal SARS-CoV-2 infection status, were obtained by following up the patients within one month after discharge. The study was approved by the Institutional Review Board of the Shandong Provincial Maternal and Child Health Care Hospital Affiliated to Qingdao University. The personal information of all study participants was anonymized prior to statistical analysis.

### 2.2 Patient selection and cohorts

**Fig. 1:**
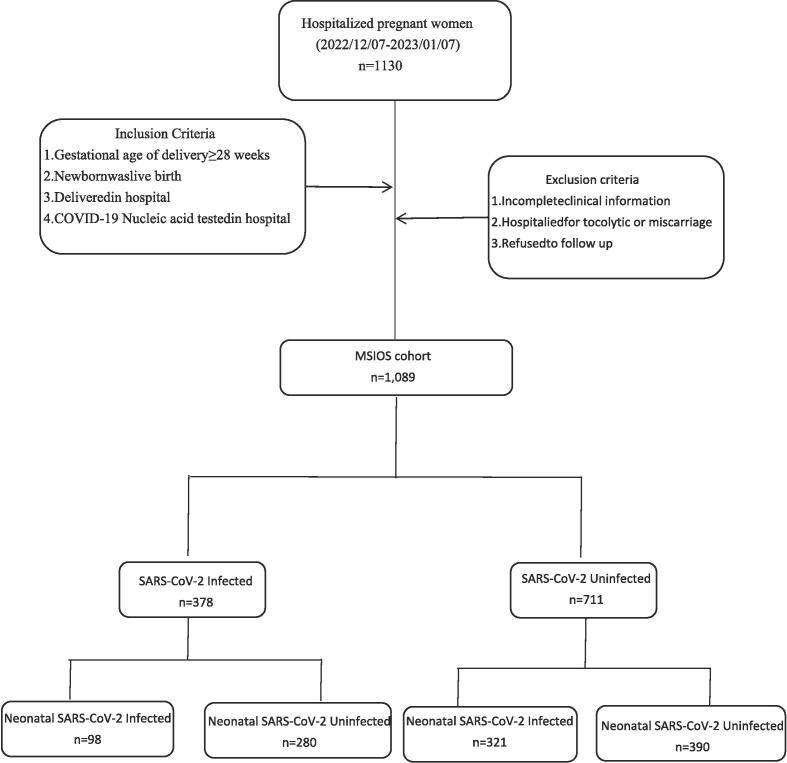
The algorithm of study subject selection. Abbreviation: MSIOS, The cohort registered and named as Maternal SARS-CoV-2 Infection and Offspring Susceptibility.

This study included a cohort of admitted pregnant women for delivery, and all study subjects were enrolled if they（a）The gestational age of delivery was more than 28 weeks,（b）The newborn was live birth,（c）Delivered in the hospital (natural delivery or Caesarean section),（d）COVID-19 Nucleic acid test was performed in the hospital (recorded positive polymerase chain reaction test negative or positive). If the pregnant women had incomplete clinical information, hospitalized for preventing miscarriage therpay or miscarriage, and refused to follow up would be excluded. All pregnant women with previous COVID-19 infections were also be excluded. The infection of SARS-CoV-2 was determined by nucleic acid test, with or without the presence of clinical symptoms such as fever, cough, shortness of breath or difficulty breathing, fatigue, muscle or body aches, headache, new loss of taste or smell, and sore throat. Finally, 1089 cases were included in the study and divided into two groups according to the infectious status of SARS-CoV-2 (Fig. 1).

### 2.3 Covariates and outcomes

All covariates were determined and included from variables that may influence maternal and fetal health, and were considered from three aspects of maternal, paternal, and neonatal. Maternal factors included age, BMI, education level, prenatal vaccination status, folic acid supplementation, medication use during pregnancy, age at first pregnancy and menarche, the number of parturitions, history of preterm birth or abortion, menstrual cycle regularity, gestational age at delivery, delivery mode, and conception method. Paternal factors included age, BMI, cigarette and alcohol consumption, and education level. Neonatal factor was the sex of the newborn. The primary outcome of the study was neonatal infection with SARS-CoV-2, and the secondary outcomes were delivery pattern and gestational age at delivery.

### 2.4 Statistical analysis

All data were collected using Microsoft Excel 2019 then visualized and statistically analyzed using R version 4.3.0 for Windows. Baseline characteristics were described as median with interquartile range for continuous variables and frequency with proportion for categorical variables. A logistic regression model was established to predict the risk of neonatal infection with SARS-CoV-2. A two-tailed p-value < 0.05 was considered statistically significant.

## 3 Results

### 3.1 Demographic characteristics of study subjects

A total of 1089 hospitalized pregnant women from November 7,2022 to January 7,2023 were included in this cohort. of these, 378 were diagnosed with COVID-19 and 711 were not (Table 1). Compared to the uninfected group, the infected pregnant women had lower rate of folic acid supplementation. The pregnancy body mass index, maternal vaccination rate, pregnancy medication in the infected group were higher than that in the uninfected group. The differences of other indicators were of no statistical significance between these two groups.

**Table 1:** Baseline characteristics of study pregnant women and comparison between the SARS-CoV-2 infected and uninfected groups.

	ALL (n = 1089)	Uninfected (n = 711)	Infected Control group (n = 378)	*p* value
**Maternal factors**				
Age at pregnancy (yrs)	31.00 [28.00;33.00]	30.00 [28.00;33.00]	31.00 [28.00;34.00]	0.359
Pregnancy BMI (kg/m^2^)	26.73 [24.09;29.36]	26.64 [23.73;29.22]	26.87 [24.61;29.57]	0.048
Education background (n)				0.092
High school and the following degree	130(11.94 %)	94(13.22 %)	36(9.53 %)	
University Graduate	806 (74.01 %)	515 (72.43 %)	291 (76.98 %)	
Postgraduate	153 (14.05 %)	102 (14.35 %)	51 (13.49 %)	
SARS-CoV-2 infection (n)				−
Infected	378 (34.71 %)	−	−	
Uninfected	711(65.29 %)	−	−	
Prenatal vaccination (n)				0.004
Yes	846 (77.69 %)	533 (74.96 %)	313(82.80 %)	
No	243 (22.31 %)	178 (25.04 %)	65(17.20 %)	
Folic acid supplementation (n)				0.001
Yes	1026 (94.21 %)	683 (96.06 %)	343 (90.74 %)	
No	63 (5.79 %)	28 (3.94 %)	35 (9.26 %)	
Pregnancy medication(n)				0.040
Yes	91 (8.36 %)	58 (8.22 %)	38 (9.92 %)	
No	998(91.64 %)	648 (91.78 %)	345 (90.08 %)	
Age at first pregnancy (yrs)	28.00 [25.00;30.00]	28.00 [25.00;30.00]	27.00 [25.00;29.75]	0.328
Delivery history				0.643
0	441 (40.50 %)	292 (41.07 %)	149 (39.42 %)	
1	648 (59.50 %)	419 (58.93 %)	229 (60.58 %)	
History of preterm birth (n)				0.092
0	1069 (98.16 %)	702 (98.73 %)	367 (97.09 %)	
≥1	20 (1.84 %)	9 (1.27 %)	11 (2.91 %)	
History of Abortion (n)				0.927
0	1029 (94.49 %)	671 (94.37 %)	358 (94.71 %)	
≥1	60 (5.51 %)	40 (5.63 %)	20 (5.29 %)	
Age at menarche (yrs)	14.00 [14.00;14.00]	14.00 [14.00;14.00]	14.00 [14.00;14.00]	0.037
Menstrual days (days)	6.00 [5.50;6.00]	6.00 [5.50;6.00]	6.00 [5.50;6.00]	0.743
Menstrual cycle days (days)	30.00 [30.00;30.00]	30.00 [30.00;30.00]	30.00 [30.00;30.00]	0.425
Menstrual cycle regularity (n)				0.142
Regular	1013 (93.02 %)	655 (92.12 %)	358 (94.71 %)	
Irregular	76 (6.98 %)	56 (7.88 %)	20 (5.29 %)	
Conception method (n)				0.986
Artificial technology	56 (5.14 %)	36 (5.06 %)	20 (5.29 %)	
Natural pregnancy	1033 (94.86 %)	675 (94.94 %)	358 (94.71 %)	
**Paternal factors**				
Age	30.00 [27.00;34.00]	30.00 [27.00;34.00]	30.00 [27.00;34.00]	0.234
BMI	24.80 [22.86;27.17]	24.84 [23.01;26.99]	24.67 [22.61;27.70]	0.697
Smoke consumption(n)				0.420
Yes	303 (27.82 %)	204 (28.69 %)	99 (26.19 %)	
No	786 (72.18 %)	507 (71.31 %)	279 (73.81 %)	
Alcohol consumption (n)				0.492
Yes	417 (38.29 %)	278 (39.10 %)	139 (36.77 %)	
No	672 (61.71 %)	433 (60.90 %)	239 (63.23 %)	
Education background (n)				0.798
High school and the following degree	196 (18.00 %)	133(18.70 %)	63 (16.67 %)	
University Graduate	746 (68.50 %)	480 (67.51 %)	266 (70.37 %)	
Postgraduate	147 (13.50 %)	98 (13.78 %)	49 (12.96 %)	

Abbreviations: BMI, body mass index. Values reported for continuous variables are medians with interquartile ranges (IQR).

### 3.2 Primary and secondary outcomes

Compared to the uninfected group, the rate of neonatal infection with SARS-CoV-2 was lower in the infected group, with 321 cases in the uninfected group and 98 cases in the infected group, accounting for 45.15 % and 25.93 %, respectively(Table 2). There were no statistically significant differences between the infected and uninfected groups with regard to the rate of vaginal delivery or gestational age at delivery. Also, no significant differences were observed between paternal factors and other neonatal outcomes.

**Table 2:** The outcomes of the maternal and neonatal between the SARS-CoV-2 infected and uninfected groups.

	ALL (n = 1089)	Uninfected (n = 711)	Infected Control group (n = 378)	*P* value
**Delivery outcomes**				
Delivery weeks	39.29 [38.57;40.14]	39.29 [38.50;40.14]	39.29 [38.57;40.14]	0.898
Delivery mode (n)				0.626
Vaginal delivery	626 (57.48 %)	413 (58.09 %)	213 (56.35 %)	
Cesarean delivery	463 (42.52 %)	298 (41.91 %)	165 (43.65 %)	
**Neonatal factors**				
SARS-CoV-2 infection (n)				<0.001
Infected	419 (38.48 %)	321 (45.15 %)	98 (25.93 %)	
Uninfected	670 (61.52 %)	390 (54.85 %)	280 (74.07 %)	
Sex assigned at birth (n)				1.000
Male	556 (51.06 %)	363 (51.05 %)	193 (51.06 %)	
Female	533 (48.94 %)	348 (48.95 %)	185 (48.94 %)	
Birth weight	3300.00 [3020.00;3550.00]	3290.00 [3000.00;3560.00]	3300.00 [3022.50;3550.00]	0.477

### 3.3 Logistic regression analysis of risk factors for neonatal SARS-CoV-2 infection

The results of the logistic regression analysis(Table 3) showed that there was an increased risk of neonatal SARS-CoV-2 infection rate regarding the pregnancy BMI (OR 95 %CI: 1.04[1.01, 1.08]), age at first pregnancy (OR 95 %CI: 1.05[1.01, 1.10]), age at menarche (OR 95 %CI: 1.13[1.02, 1.26]) and parturition（Yes vs. No） (OR 95 %CI:1.4 [1.04,1.88]), while the decreased risk for neonatal infection was determined in maternal vaccination (OR 95 %CI:0.63[0.46, 0.85]) and maternal infection of SARS-CoV-2 (OR 95 %CI: 0.45[0.34, 0.60]).

**Table 3:** Logistic regression analysis of neonatal infection with SARS-CoV-2.

Variables	crude OR(95 %CI)	adj. OR(95 %CI)	P value
Age at pregnancy (yrs)	1 (0.97,1.03)	0.98 (0.94,1.02)	0.27
Pregnancy BMI (kg/m^2^)	1.04 (1.01,1.07)	1.04 (1.01,1.08)	0.011
Vaccination			
Yes vs. No	0.61 (0.46,0.82)	0.63 (0.46,0.85)	0.003
Age at menarche (yrs)	1.17 (1.06,1.29)	1.13 (1.02,1.26)	0.009
Parturition			
Yes vs. No	1.27 (0.99,1.63)	1.4 (1.04,1.88)	0.026
Menstrual days	1.05 (0.91,1.21)	1.08 (0.93,1.26)	0.315
Age at first pregnancy (yrs)	1.02 (0.99,1.06)	1.05 (1.01,1.1)	0.025
Menstrual cycle days	0.995 (0.98,1.01)	0.9913 (0.98,1.00)	0.1
Conception method			
Natural vs. Artificial	1.23 (0.7,2.17)	1.28 (0.69,2.38)	0.436
Folic acid supplementation			
Yes vs. No	1.3682 (0.79,2.36)	1.0022 (0.56,1.78)	0.994
Pregnancy medication:			
Yes vs. No	0.77 (0.49,1.21)	0.76 (0.47,1.23) y	0.253
Maternal SARS-CoV-2 infection			
Infected vs. Uninfected	0.43 (0.32,0.56)	0.45 (0.34,0.6)	< 0.001
GDM			
Yes vs. No	0.96 (0.74,1.27)	1.02 (0.77,1.37)	0.872
Paternal age	0.99 (0.96,1.01)	0.98 (0.95,1)	0.077
Paternal smoking			
Yes vs. No	1.24 (0.95,1.63)	1.31 (0.95,1.8)	0.096
Paternal drinking			
Yes vs. No	1.04 (0.81,1.34)	0.95 (0.71,1.26)	0.705
Paternal BMI (kg/m^2^)	0.9951 (0.96,1.03)	0.994 (0.96,1.03)	0.737
Preterm Birth			
Yes vs. No	1.01 (0.56,1.82)	1.17 (0.63,2.19)	0.615

Abbreviation: GDM, gestational diabetes mellitus, BMI, body mass index.

## 4 Discussion

During the COVID-19 pandemic, it has been a critical issue on how to protect young children from infection. Previous studies have suggested several strategies for reducing the risk of infection among young children, including vaccination of eligible household members, avoiding large crowds and maintaining physical distance, wearing masks in indoor public spaces or crowded outdoor settings, practicing good hand hygiene, and staying home when sick. However, the protective effects of maternal infection on neonates remain a subject of debate.

In this study, we found that maternal vaccination or perinatal infection with COVID-19 had protective effects on the newborn. These findings are consistent with a study conducted in Israel with 1094 participants, which showed that antenatal BNT162b2 mRNA vaccination elicited a strong maternal humoral IgG response that crossed the maternal-fetal interface and approached maternal titers in the fetus within 15 days following the first dose. Furthermore, the ratio of maternal to neonatal anti-COVID-19 antibodies did not differ when comparing sensitization (vaccine vs. infection)[13]. Similar studies indicated that COVID-19 vaccination during pregnancy can produce functional IgG antibodies in maternal circulation, which had been detected in cord blood at birth, and can protect newborns and infants from COVID-19 infection [14], [15], [13]. A study published by the JAMA Pediatrics manifested that the COVID-19 antibody in mother can not only be transmitted to the baby through the maternal fetal barrier, but also through breastfeeding[16]. There was a significant and positive correlation between maternal serum levels of SARS-Cov-2 IgG and cord blood (R = 0.483, p = 0.0001), neonatal blood spot (R = 0.515, p = 0.004), and breast milk levels (R = 0.396, p = 0.005) of SARS-CoV-2 IgG[17]. During breastfeeding, IgA, IgG and IgM confer neonatal mucosal immune protection by binding to commensal and pathogenic microbes and their virulence factors to mediate immune exclusion and neutralization [18].

Comparison of vaccinated with convalescent COVID-19 patients revealed significantly increased SARS −CoV-2 IgG levels in maternal serum and cord blood among vaccinated women (p < 0.001) [17]. Therefore, vaccination of pregnant women was an effective protective method for newborns to from infection. FIGO considered “there are no risks—actual or theoretical—that would outweigh the potential benefits of vaccination for pregnant women. We support offering COVID-19 vaccination to pregnant and breastfeeding women” [19]. Data from more than 180,000 vaccinated persons show that immunization against COVID-19 with an mRNA vaccine is safe for pregnant women [20]. Also, Vaccination of pregnant people reduces the COVID-19-related increase in maternal or fetal morbidity [21]. An interesting finding in our study was that the vaccination rate of pregnant women infected with COVID-19 was higher than that of the uninfected group, accounting for 37 % in infected group and 26.7 % in uninfected group, which seems contradictory. However, there may be three reasons. Firstly, the higher infection rate in vaccinated women compared to unvaccinated women could be due to a higher proportion of previously infected women with naturally acquired immunity in the latter group, women with a prior infection are more likely to have not received a COVID-19 vaccine because protected by natural immunity. Secondly, according to China's national conditions during the COVID-19 pandemic, pregnant women were not recommended to be vaccinated. Moreover, different doses of vaccination may produce different immune results. Lastly, the pregnant women who were not vaccinated had stronger sense of self-protection. In order to avoid infection, they may pay more attention to their hand hygiene and quarantine themselves conscientiously.

It is still inconsistent whether COVID-19 can affect the pattern of pregnancy delivery[22], [23], [24], [25], [8]. Although COVID-19 was not the indication of caesarean section, most studies showed that caesarean section was the primary choice of delivery for COVID-19 patients[22], [23]. Consistently, some studies reported similar findings with us[24], [8], where the rate of caesarean section in the infected group was higher as compared to the control, but we did not find statistical significances. Some studies focused on the impact of COVID-19 on preterm delivery, insisting that COVID-19 would lead to an increase in preterm delivery rate [24], [7]. The delivery weeks in our study had no statistical differences which was 39.29 [38.50;40.14] in the uninfected group and 39.29 [38.57;40.14] in the infected group.

This study has several strengths as it used information from parents to study the susceptibility of newborns to SARS-CoV-2 for the first time. The valuable information includes mother's Body mass index, mother's delivery history and menarche age which may provide some useful information for reducing the susceptibility of newborns. Also, there are several limitations in the study. Firstly, most of the pregnant women in this cohort were infected in the third trimester, and the extended studies need to include cases in the first and second trimester which make the study more comprehensive. Secondly, it is a single center study although the number of cases in this study exceeds one thousand. Further research needs to incorporate multi-center studies to make them more convincing. Thirdly, the limitation of this retrospective cohort study lies in the lack of prospective standardization in the detection, diagnosis, and management of COVID-19. The information collected through telephone interview may have introduced a desirability bias, such as the infections of newborns and father's habits that may be perceived as undesirable behaviours (e.g., smoking or alcohol consumption). Due to the reliance of this study on medical record data, there may be inconsistencies in the COVID-19 testing protocol, diagnostic criteria, and treatment methods between the entire study period and patients. The lack of standardized prospective testing, diagnosis, and treatment plans may lead to bias or affect observation results. A prospective study with predefined protocols will help standardize these factors and provide stronger evidence for the protective effects of COVID-19 infection in pregnant women and vaccination on neonatal susceptibility.

In conclusion, maternal vaccination (OR 95 %CI:0.63[0.46, 0.85]) or maternal infection with SARS-CoV-2 (OR 95 %CI: 0.45[0.34, 0.60]) can reduce the susceptibility of the newborn in the infected group. These findings provide strong evidence to support the vaccination of pregnant women against COVID-19, which can benefit not only the mother but the newborn by reducing the risk of infection.

**Ethical approval statement**

The study was approved by the Institutional Review Board of the Shandong Provincial Maternal and Child Health Care Hospital Affiliated to Qingdao University. The personal information of all study participants was anonymized prior to statistical analysis.

**Funding**

This study was supported by the Taishan Scholar Program of Shandong Province (No. tsqn202211359).

## CRediT authorship contribution statement

**Xiao-Dan Zhu:** Writing – review & editing, Writing – original draft. **Yan-Jie Peng:** Investigation, Formal analysis, Data curation. **Ying Chen:** Investigation, Data curation. **Mei Xue:** Investigation, Data curation. **Ai-Juan Zhang:** Investigation, Formal analysis. **Yu Peng:** Investigation, Formal analysis, Data curation. **Rong Mei:** Formal analysis, Data curation. **Mei-Rong Tian:** Investigation, Data curation. **Lin Zhang:** Writing – review & editing, Supervision, Project administration, Conceptualization.

## Declaration of competing interest

The authors declare that they have no known competing financial interests or personal relationships that could have appeared to influence the work reported in this paper.

## Figures and Tables

**Table 4:** Logistic regression analysis to predict preterm birth, birth weight, and delivery mode*.

Variables	Preterm birth (Yes vs. No)			Birth weight (≥2500 g vs. < 2500 g)			Delivery mode (Caesarean vs. vaginal)		
	crude OR(95 %CI)	adj. OR(95 %CI)	P	crude OR(95 %CI)	adj. OR(95 %CI)	P	crude OR(95 %CI)	adj. OR(95 %CI)	P
Maternal SARS-CoV-2 infection									
Infected vs. Uninfected	0.91 (0.49,1.68)	0.99 (0.52,1.89)	0.98	1.52 (0.83,2.78)	1.49 (0.8,2.76)	0.20	1.07 (0.83,1.38)	1.02 (0.78,1.32)	0.90
Age at pregnancy (yrs)	1.03 (0.96,1.11)	1.10 (1.00,1.20)	0.05	1.04 (0.97,1.11)	1.00 (0.92,1.09)	0.94	1.05 (1.02,1.08)	1.07 (1.03,1.11)	< 0.01
Pregnancy BMI (kg/m^2^)	0.97 (0.91,1.05)	0.98 (0.91,1.06)	0.67	1.00 (0.93,1.06)	0.98 (0.92,1.05)	0.64	1.06 (1.03,1.09)	1.05 (1.02,1.08)	< 0.01
Age at menarche (yrs)	0.99 (0.83,1.19)	1.05 (0.85,1.3)	0.68	0.98 (0.85,1.14)	0.97 (0.83,1.13)	0.69	1.00 (0.93,1.08)	0.99 (0.92,1.07)	0.88
Parturition									
Yes vs. No	0.38 (0.21,0.68)	0.27 (0.14,0.54)	<0.01	1.56 (0.91,2.66)	1.67 (0.91,3.06)	0.10	1.18 (0.92,1.51)	0.91 (0.69,1.21)	0.53
Menstrual days	0.95 (0.68,1.31)	0.91 (0.65,1.28)	0.60	1.08 (0.80,1.45)	1.09 (0.80,1.48)	0.60	0.80 (0.69,0.91)	0.83 (0.72,0.96)	0.01
Age at first pregnancy (yrs)	0.99 (0.92,1.07)	0.91 (0.83,1.00)	0.05	1.05 (0.97,1.13)	1.06 (0.97,1.15)	0.22	0.98 (0.95,1.02)	0.95 (0.91,0.99)	0.01
Menstrual cycle days	0.97 (0.91,1.03)	0.96 (0.9,1.02)	0.07	1.01 (0.98,1.04)	1.01 (0.98,1.05)	0.29	1.00 (0.99,1.01)	1.00 (0.99,1.01)	0.62
Conception method									
Natural vs. Artificial	0.25 (0.11,0.56)	0.21 (0.08,0.52)	0.00	2.31 (0.95,5.64)	2.71 (1.04,7.03)	0.06	0.54 (0.31,0.92)	0.59 (0.33,1.05)	0.07
Folic acid supplementation:									
Yes vs. No	5800195.03 (0,Inf)	5179385.13 (0,Inf)	0.02	0.9 (0.27,2.96)	1.05 (0.31,3.57)	0.94	0.86 (0.52,1.43)	0.95 (0.55,1.62)	0.84
Pregnancy medication:									
Yes vs. No	0.22 (0.03,1.61)	0.19 (0.02,1.46)	0.04	1.68 (0.51,5.48)	1.88 (0.56,6.28)	0.26	0.97 (0.63,1.49)	0.89 (0.57,1.41)	0.63
GDM									
Yes vs. No	1.01 (0.54,1.9)	0.88 (0.45,1.74)	0.72	1.36 (0.72,2.56)	1.42 (0.73,2.72)	0.29	1.19 (0.92,1.56)	1.03 (0.78,1.36)	0.82
Paternal age	1.01 (0.96,1.06)	1.00 (0.94,1.06)	0.90	1.02 (0.97,1.07)	1.00 (0.95,1.06)	0.94	1.01 (1.00,1.04)	1.00 (0.98,1.03)	0.96
Paternal smoking									
Yes vs. No	0.65 (0.32,1.33)	0.48 (0.22,1.07)	0.06	0.76 (0.43,1.34)	0.96 (0.5,1.84)	0.90	1.04 (0.8,1.36)	0.96 (0.71,1.31)	0.80
Paternal drinking									
Yes vs. No	1.45 (0.82,2.58)	1.7 (0.9,3.24)	0.11	0.73 (0.43,1.24)	0.73 (0.4,1.34)	0.32	1.11 (0.87,1.42)	1.09 (0.83,1.44)	0.54
Paternal BMI (kg/m^2^)	0.97 (0.9,1.05)	0.96 (0.88,1.05)	0.36	1.01 (0.94,1.09)	1.02 (0.94,1.1)	0.69	1.02 (0.99,1.05)	1.01 (0.98,1.05)	0.43

* Maternal SARS-CoV-2 infection status was included as the primary exposure factor.

**Table 5:** Logistic regression analysis to predict preterm birth, birth weight, and delivery mode*.

Variables	Preterm birth (Yes vs. No)			Birth weight (≥2500 g vs. < 2500 g)			Delivery mode (Caesarean vs. vaginal)		
	crude OR(95 %CI)	adj. OR(95 %CI)	P	crude OR(95 %CI)	adj. OR(95 %CI)	P	crude OR(95 %CI)	adj. OR(95 %CI)	P
Vaccination									
Yes vs. No	1.29 (0.62,2.70)	1.37 (0.64,2.96)	0.41	0.92 (0.48,1.78)	0.91 (0.47,1.78)	0.79	1.01 (0.75,1.34)	1.08 (0.8,1.46)	0.60
Age at pregnancy (yrs)	1.03 (0.96,1.11)	1.10 (1.00,1.20)	0.05	1.04 (0.97,1.11)	1.00 (0.92,1.10)	0.92	1.05 (1.02,1.08)	1.07 (1.03,1.11)	< 0.01
Pregnancy BMI (kg/m^2^)	0.97 (0.91,1.05)	0.98 (0.91,1.06)	0.65	1.00 (0.93,1.06)	0.99 (0.92,1.06)	0.73	1.06(1.03,1.06)	1.05 (1.02,1.08)	0.00
Age at menarche (yrs)	0.99 (0.83,1.19)	1.06 (0.85,1.31)	0.63	0.98 (0.85,1.14)	0.96 (0.82,1.12)	0.60	1.00 (0.93,1.08)	1.00 (0.92,1.07)	0.90
Parturition									
Yes vs. No	0.38 (0.21,0.68)	0.27 (0.14,0.54)	< 0.01	1.56 (0.91,2.66)	1.65 (0.90,3.04)	0.11	1.18 (0.92,1.51)	1.18 (0.92,1.51)	0.53
Menstrual days	0.95 (0.68,1.31)	0.91 (0.64,1.27)	0.57	1.08 (0.8,1.45)	1.1 (0.81,1.50)	0.56	1.18 (0.92,1.51)	0.83 (0.72,0.96)	< 0.01
Age at first pregnancy (yrs)	0.99 (0.92,1.07)	0.91 (0.84,1.00)	0.05	1.05 (0.97,1.13)	1.05 (0.97,1.15)	0.24	1.00 (0.93,1.08)	0.99 (0.92,1.07)	0.90
Menstrual cycle days	0.97 (0.91,1.03)	0.96 (0.90,1.02)	0.08	1.01 (0.98,1.04)	1.01 (0.98,1.05)	0.31	1.00 (0.99,1.01)	1.00 (0.99,1.01)	0.62
Conception method									
Natural vs. Artificial	0.25 (0.11,0.56)	0.20 (0.08,0.51)	0.00	2.31 (0.95,5.64)	2.72 (1.05,7.08)	0.06	0.54 (0.31,0.92)	0.58 (0.33,1.04)	0.07
Folic acid supplementation:									
Yes vs. No	5800195.03 (0,Inf)	5256996.65 (0,Inf)	0.02	0.90 (0.27,2.96)	0.95 (0.28,3.22)	0.94	0.86 (0.52,1.43)	0.94 (0.55,1.6)	0.82
Pregnancy medication:									
Yes vs. No	0.22 (0.03,1.61)	0.19 (0.02,1.50)	0.05	1.68 (0.51,5.48)	1.92 (0.57,6.41)	0.25	0.97 (0.63,1.49)	0.9 (0.57,1.42)	0.66
GDM									
Yes vs. No	1.01 (0.54,1.90)	0.88 (0.45,1.73)	0.71	1.36 (0.72,2.56)	1.41 (0.74,2.72)	0.29	1.19 (0.92,1.56)	1.03 (0.78,1.36)	0.84
Paternal age	1.01 (0.96,1.06)	1.00 (0.94,1.06)	0.88	1.02 (0.97,1.07)	1.00 (0.95,1.06)	0.90	1.02 (1.00,1.04)	1.00 (0.98,1.03)	0.96
Paternal smoking									
Yes vs. No	0.65 (0.32,1.33)	0.48 (0.22,1.06)	0.06	0.76 (0.43,1.34)	0.95 (0.49,1.82)	0.87	1.04 (0.8,1.36)	0.96 (0.71,1.31)	0.80
Paternal drinking									
Yes vs. No	1.45 (0.82,2.58)	1.69 (0.89,3.21)	0.11	0.73 (0.43,1.24)	0.74 (0.40,1.34)	0.32	1.11 (0.87,1.42)	1.09 (0.82,1.44)	0.54
Paternal BMI (kg/m^2^)	0.97 (0.90,1.05)	0.96 (0.88,1.05)	0.36	1.01 (0.94,1.09)	1.02 (0.94,1.10)	0.69	1.02 (0.99,1.05)	1.01 (0.98,1.05)	0.43

* Vaccination was included as the primary exposure factor.

## Data Availability

Data will be made available on request.
